# Radium-223 in asymptomatic patients with castration-resistant prostate cancer and bone metastases treated in an international early access program

**DOI:** 10.1186/s12885-018-5203-y

**Published:** 2019-01-07

**Authors:** Axel Heidenreich, Silke Gillessen, Daniel Heinrich, Daniel Keizman, Joe M. O’Sullivan, Joan Carles, Manfred Wirth, Kurt Miller, John Reeves, Monica Seger, Sten Nilsson, Fred Saad

**Affiliations:** 1 Department of Urology, University Hospital Cologne, Cologne, Germany; 2 Division of Cancer Sciences, University of Manchester and the Christie, Manchester, UK; 3 Medical Oncology and Haematology, Kantonsspital St Gallen, St Gallen, Switzerland; 4 University of Bern, Bern, Switzerland; 5 Department of Oncology, Akershus University Hospital, Lørenskog, Norway; 6 Genitourinary Oncology Service, Institute of Oncology, Meir Medical Center, Kfar-Saba, Israel; 7 Department of Clinical Oncology, The Centre for Cancer Research and Cell Biology, Queen’s University Belfast and the Northern Ireland Cancer Centre, Belfast, Northern Ireland, UK; 8 Department of Medical Oncology, Vall d’Hebron University Hospital, Vall d’Hebron Institute of Oncology, Barcelona, Spain; 9 Department of Urology, University Hospital Carl-Gustav Carus, Dresden, Germany; 10 Department of Urology, Charité University Medicine Berlin, Berlin, Germany; 11 Pharmaceutical Division of Bayer, Whippany, NJ USA; 12 Department of Oncology, Karolinska University Hospital, Stockholm, Sweden; 13 Department of Urology, Centre Hospitalier de l’Université de Montréal (CHUM), Montreal, QC Canada

**Keywords:** Radium-223, mCRPC, asymptomatic, Symptomatic, Bone metastases

## Abstract

**Background:**

Radium-223, a targeted alpha therapy, is used to treat symptomatic patients with castration-resistant prostate cancer (CRPC) and bone metastases. Data for radium-223 in asymptomatic CRPC patients with bone metastases are lacking.

**Methods:**

This was a prospective, single-arm phase 3b study. Patients with metastatic CRPC (malignant lymphadenopathy not exceeding 6 cm was allowed, visceral disease was excluded) received radium-223, 55 kBq/kg intravenously, every 4 weeks for up to 6 cycles. Co-primary endpoints were safety and overall survival. Post hoc analyses were performed according to baseline asymptomatic or symptomatic disease status. Asymptomatic status was defined as no pain and no opioid use at baseline.

**Results:**

Seven hundred eight patients received ≥1 radium-223 injection: 548 (77%) were symptomatic to various degrees, and 135 (19%) were asymptomatic. Asymptomatic patients had more favorable baseline disease characteristics than symptomatic. A lower proportion of asymptomatic versus symptomatic patients had received prior abiraterone (25% vs 35%) and prior docetaxel (52% vs 62%). A higher proportion of asymptomatic (71%) versus symptomatic (55%) patients completed radium-223 treatment. Overall survival (hazard ratio [HR] 0.486), time to disease progression (HR 0.722) and time to first symptomatic skeletal event (HR 0.328) were better in asymptomatic than symptomatic patients. Alkaline phosphatase (ALP) response rates were similar (46% vs 47%), and ALP normalization (44% vs 25%) and prostate-specific antigen response rates (21% vs 13%) were higher in asymptomatic than symptomatic patients. A lower proportion of asymptomatic patients reported treatment-emergent adverse events (TEAEs, 61% vs 79%), grade 3–4 TEAEs (29% vs 40%) and drug-related TEAEs (28% vs 44%). There were two treatment-related deaths, both in patients with baseline symptomatic disease.

**Conclusions:**

Using radium-223 earlier in the disease course, when patients are asymptomatic or minimally symptomatic, may enable patients to complete treatment and optimize treatment outcome compared to symptomatic patients, and therefore may allow sequencing with other life-prolonging therapies.

**Trial registration:**

The study was registered with ClinicalTrials.gov, number NCT01618370 on June 13, 2012 and the European Union Clinical Trials Register, EudraCT number 2012–000075-16 on April 4, 2012.

**Electronic supplementary material:**

The online version of this article (10.1186/s12885-018-5203-y) contains supplementary material, which is available to authorized users.

## Background

Radium-223 dichloride (radium-223), a targeted alpha therapy, is incorporated into newly formed bone in areas of osteoblast activity and increased bone turnover surrounding prostate cancer bone metastases [1, 2]. Radium-223 emits high energy alpha particles over a short range resulting in a localized potent antitumor effect and inhibition of tumor-induced osteoblastic activity in preclinical models [3].

Radium-223 is recommended for the treatment of patients with mCRPC and symptomatic bone metastases [4, 5]. In the pivotal phase 3 ALSYMPCA study, patients with mCRPC and symptomatic bone metastases assigned to radium-223 with best standard of care (BSoC) demonstrated prolonged overall survival (median 14.0 vs 11.2 months, hazard ratio [HR] 0.70; 95% CI, 0.58–0.83; *p* < 0.001) and delayed time to first symptomatic skeletal event (SSE) compared with those assigned to placebo with BSoC [6, 7]. Radium-223 was generally well tolerated and improvements in patient quality of life were reported compared with patients treated in the placebo arm [6, 8].

In the phase 3 study patients with symptomatic bone metastases were defined as those who regularly used analgesic medication (opioids or non-opioids were allowed), or those who were pain-free, but had received external beam radiation therapy (EBRT) for cancer-related bone pain within a 12 week period before enrollment [6]. However, patients with mCRPC and bone predominant disease often initially present without symptoms [9] and data for radium-223 in these patients are lacking.

Data from clinical trials investigating life-prolonging agents have shown that treating patients with mCRPC earlier in their disease course, for example those who are mildly symptomatic or asymptomatic, can be beneficial. In the TAX 327 study docetaxel every 3 weeks compared with mitoxantrone demonstrated an overall survival benefit (HR 0.76) in patients with mCRPC, which was also demonstrated in a subgroup analysis of patients without pain at baseline (HR 0.73) [10]. Androgen receptor axis-targeted agents, abiraterone acetate (abiraterone, HR 0.65) [11] and enzalutamide (HR 0.63) [12], provided a significant survival benefit in comparison with placebo in patients with mCRPC progressing on docetaxel. A similar overall survival benefit was reported in subgroup analyses of patients with no clinically significant baseline pain (abiraterone HR 0.64, and enzalutamide HR 0.59). Moreover, abiraterone (HR 0.81) and enzalutamide (HR 0.71) demonstrated a survival advantage over placebo when administered first-line in asymptomatic or mildly symptomatic patients with mCRPC [13, 14]. Similarly, it may also be the case that radium-223 is beneficial to patients with asymptomatic bone predominant metastases.

In a phase 3b international early access program (iEAP), patients with asymptomatic mCRPC were included [15], which enabled the current analysis investigating the safety and activity of radium-223 in this patient population.

## Methods

### Study design and treatment

Study design and patient inclusion and exclusion criteria for this phase 3b study have been previously reported in detail [15]. Patients were ≥ 18 years or older, had histologically or cytologically confirmed progressive bone-predominant mCRPC with two or more skeletal metastases on imaging (symptomatic or asymptomatic), and no visceral disease (lymph node-only metastases not exceeding 6 cm were allowed) [15].

Patients were treated with intravenous injections of radium-223, 55 kBq/kg, every 4 weeks for up to 6 cycles [15]. Concomitant treatment was permitted including abiraterone or enzalutamide, and bone supportive agents as previously described [15]. Supportive care was delivered according to local institutional guidelines [15].

### Study assessments

Primary endpoints were safety and overall survival. Symptomatic disease was defined as having pain, or using opioids for cancer related pain at baseline. Asymptomatic disease was defined as no pain (Brief Pain Inventory Short Form [BPI-SF] score of 0) and no opioid use at baseline (use of non-opioid analgesics was allowed)*.* Pain severity was assessed using the self-administered validated BPI-SF questionnaire as previously described [15]. Exploratory efficacy variables included time from start of therapy to first SSE, time to disease progression, and alkaline phosphatase (ALP) response and normalization and prostate specific antigen (PSA) response.

SSEs were defined as the use of EBRT to relieve skeletal symptoms, or the occurrence of new symptomatic pathological bone fractures (vertebral or non-vertebral), or the occurrence of spinal cord compression, or a tumor-related orthopedic surgical intervention. Time to first SSE was defined as time in months from the start of radium-223 until occurrence of the first SSE during the study period. Time to disease progression was defined as the time in months from the start of radium-223 to the date that disease progression (including radiographic, clinical and PSA progression) was assessed as per the local standard of care. Total-ALP and PSA responses, and ALP normalization, were defined as previously reported [15]. Specifically responses were defined as ≥30% reduction of the blood level, compared to the baseline value, confirmed by a second value obtained approximately 4 or more weeks later. Total-ALP normalization was defined as the return of the total-ALP value to within the normal range at 12 weeks after the start of treatment, based on 2 consecutive measurements (at least 2 weeks apart), in patients who had their total-ALP above the upper limit of normal (ULN) at baseline [15].

Adverse events were coded using the Medical Dictionary for Regulatory Activities (MedDRA) version 17.1 and graded using the Common Terminology Criteria for Adverse Events version 4.03, as previously reported [15]. Treatment-emergent adverse events (TEAEs) were defined as those occurring on or after the date and time of administration of the first dose of study drug, or if they were present prior to the administration of the first dose of study drug and increased in severity during the study.

### Statistics

Exploratory safety and efficacy analyses were performed in patients who had received at least one dose of study drug and for whom symptom status at baseline could be defined. Kaplan-Meier methodology was used to estimate time-to event data. HRs were calculated using a Cox regression model [15]. The HR (asymptomatic vs symptomatic) was calculated using a Cox regression model.

## Results

### Patients

In this updated analysis of the iEAP, 708 patients received at least one radium-223 injection, of whom 683 patients could be defined by symptom status at baseline; 135 (19%) were asymptomatic and 548 (77%) were symptomatic. Twenty-five (4%) patients were excluded from the analysis as symptom status could not be confirmed for reasons including missing baseline pain scores or use of opioids for non-cancer-related pain. Of the asymptomatic patients, 19/135 (14%) only reported use of non-opioid analgesics at baseline.

Patients who were asymptomatic had more favorable baseline characteristics than those who were symptomatic, including Eastern Cooperative Oncology Group performance status (ECOG PS), lower PSA levels and longer time to metastases from initial diagnosis of prostate cancer (Table 1). A lower proportion of patients who were asymptomatic compared with symptomatic had received prior abiraterone (25% vs 35%) and prior docetaxel (52% vs 62%). A higher proportion of patients who were asymptomatic (71%) compared with symptomatic (55%) received all 6 planned cycles of radium-223 (Table 2). The most common reasons for treatment discontinuation in symptomatic (156/248, 63%) compared with asymptomatic (17/39, 43%) patients were those associated with disease progression [see Additional file 1].

**Table 1:** Baseline characteristics in radium-223 treated patients according to symptom status

Characteristics	Asymptomatic *N* = 135	Symptomatic *N* = 548
Age, median (range), years	73.0 (51.0–91.0)	72.0 (45.0–94.0)
Race, white	133 (99)	534 (97)
ECOG PS		
0	76 (56)	176 (32)
1	52 (39)	293 (53)
≥2	7 (5)	79 (14)
Combined Gleason score at initial diagnosis		
2–4	2 (1)	13 (2)
5–7	54 (40)	209 (38)
8–10	67 (50)	277 (51)
Missing	12 (9)	49 (9)
Alkaline phosphatase,^a^ U/L		
Median (range)	127.0 (19.0–1349.0)	168.0 (22.0–4236.0)
Prostate specific antigen,^b^ μg/L		
Median (range)	113.0 (0–3266.0)	154.6 (0–12,150.0)
Hemoglobin g/dL		
Median (range)	12.8 (8.7–15.8)	12.1 (8.4–18.0)
Time between initial diagnosis of prostate cancer and bone metastasis,^c^ months		
Median (range)	30.2 (0–237.7)	13.6 (0–252.2)
Prior use of bone supportive agents		
Bisphosphonates	11 (8)	35 (6)
Denosumab	4 (3)	10 (2)
Prior use of anticancer agents		
Docetaxel	70 (52)	339 (62)
Abiraterone	34 (25)	190 (35)
Enzalutamide	5 (4)	43 (8)

Data are number of patients (%) unless stated otherwise

^a^Asymptomatic, *n* = 133; symptomatic, *n* = 545

^b^Asymptomatic, *n* = 134; symptomatic, *n* = 543

^c^Asymptomatic, *n* = 87; symptomatic, *n* = 543

*ECOG PS* Eastern Cooperative Oncology Group performance status

**Table 2:** Number of radium-223 injections received according to symptom status

Radium-223 injections	Asymptomatic *N* = 135	Symptomatic *N* = 548
Median (range)	6 (1–6)	6 (1–6)
Only 1	4 (3)	33 (6)
Only 2	3 (2)	40 (7)
Only 3	5 (4)	59 (11)
Only 4	13 (10)	59 (11)
Only 5	14 (10)	57 (10)
All 6	96 (71)	300 (55)

Data are number of patients (%) unless stated otherwise

During the treatment period for radium-223, comparing asymptomatic with symptomatic patients, 21% vs 16% were treated with concomitant abiraterone, 6% vs 5% with enzalutamide, 18% each with bisphosphonates, and 19% v 18% with denosumab concomitantly.

### Efficacy

**Fig. 1:**
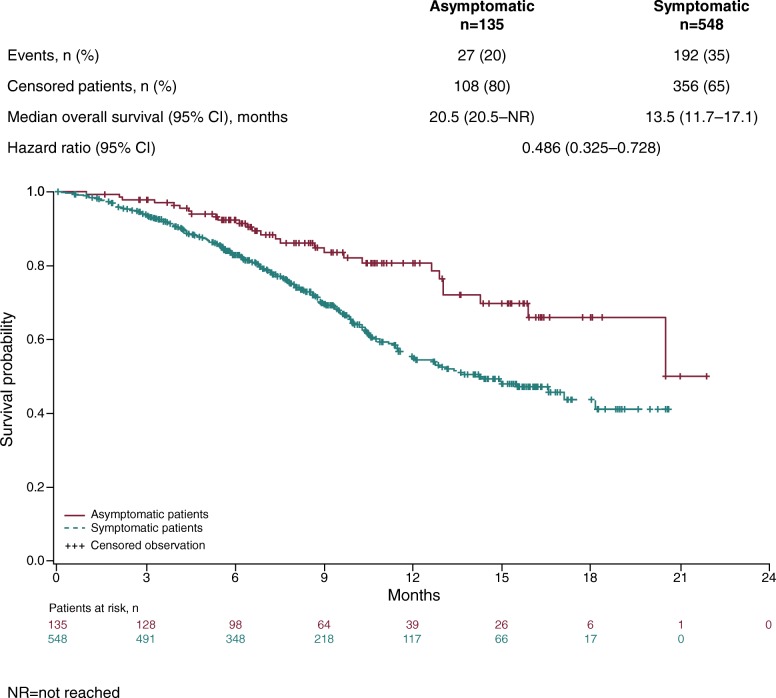
Overall survival in radium-223 treated patients according to symptom status at baseline

**Fig. 2:**
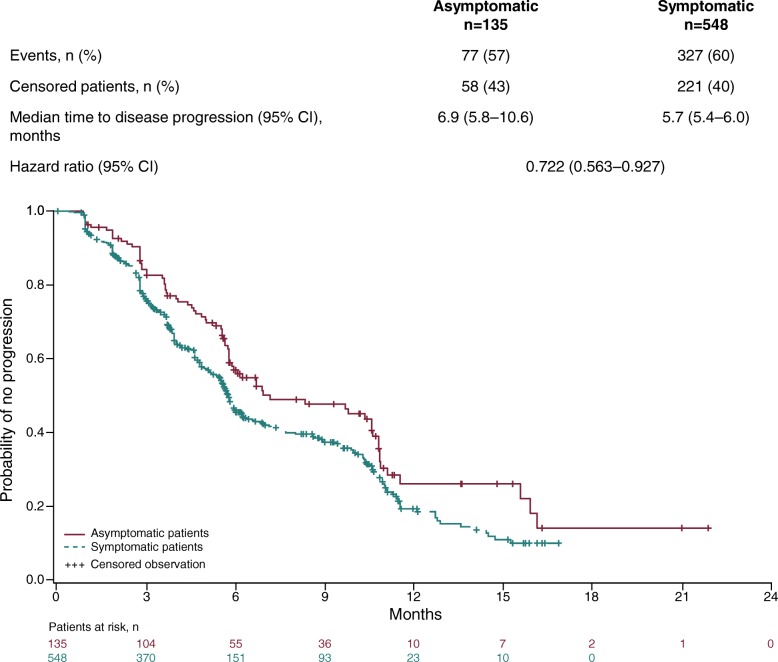
Time to disease progression in radium-223 treated patients according to symptom status at baseline

Median follow-up was 9.8 months for both asymptomatic patients (95% CI 8.5–10.9) and for symptomatic patients (95% CI 9.2–10.5). High censoring rates were noted for overall survival and time to first SSE analyses due to the short follow-up time of the trial. Longer medians for overall survival (Fig. 1) and time to disease progression (Fig. 2) were observed in asymptomatic patients compared with those who were symptomatic. HRs of 0.486 (Fig. 1) and 0.722 (Fig. 2) indicated that the risk of death or disease progression was lower by 51 and 28% respectively in the asymptomatic compared with the symptomatic group.

**Fig. 3:**
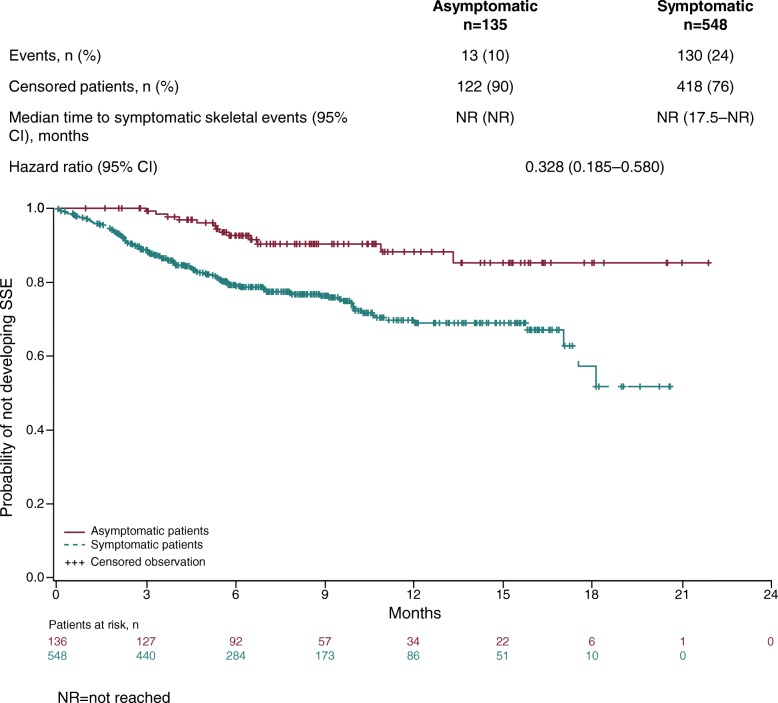
Time to first symptomatic skeletal event in radium-223 treated patients according to symptom status at baseline

During the study period, SSEs were recorded in 13/135 (10%) patients who were asymptomatic compared with 130/548 (24%) who were symptomatic at baseline [see Additional file 2]. Median time to first SSE was not reached in either of the two groups. The HR of 0.328 indicated that the risk of a SSE was lower by 67% in the asymptomatic compared with the symptomatic group (Fig. 3).

Total-ALP responses [see Additional file 3] were similar in asymptomatic (62/135, 46%) and symptomatic patients (259/548, 47%), as were total-ALP responses in those patients with baseline levels above the ULN (43/71, 61% vs 212/342, 62%). For patients with total-ALP levels above the ULN at baseline, total-ALP was more often returned to the normal range in asymptomatic patients (31/71, 44%) compared with symptomatic patients (86/342, 25%).

The PSA response rate [see Additional file 4] was higher in asymptomatic (29/135, 21%) compared with symptomatic patients (72/548, 13%), as was the PSA response rate in those patients with baseline PSA levels above the ULN (28/129, 22% vs 65/514, 13%).

### Safety

A lower proportion of asymptomatic patients (82/135, 61%) reported TEAEs compared with symptomatic patients (435/548, 79%) (Table 3). The most common grade 3–4 TEAEs (≥3% in either subgroup) in asymptomatic vs symptomatic patients were anemia, 6 (4%) vs 73 patients (13%), thrombocytopenia 2 (1%) vs 22 (4%) back pain 1 (< 1%) vs 19 (3%), bone pain, 3 (2%) vs 26 (5%) and spinal cord compression 0 vs 19 (3%) TEAEs considered to be related to treatment were reported in 38 (28%) asymptomatic patients and 243 (44%) symptomatic patients [see Additional file 5]; the most common grade 3–4 adverse event related to treatment was anemia in 3 (2%) vs 28 (5%) patients respectively. Serious adverse events were reported in 30 (22%) patients with asymptomatic and 210 (38%) patients with symptomatic disease, which in 2 (1%) and 31 patients (6%) respectively were considered to be treatment-related. Adverse events leading to permanent discontinuation were reported in 21 (16%) patients with asymptomatic disease and 123 (22%) with symptomatic disease: in 6 (4%) and 33 (4%) patients respectively they were considered to be related to treatment. Adverse events leading to death were reported in 2 (< 1%) asymptomatic patients (1 cardiac failure and 1 sepsis) and 32 (6%) symptomatic patients (most commonly due to general physical health deterioration in 12 patients). Two TEAEs leading to death were considered to be treatment-related, both in symptomatic patients (1 patient with neutropenia, and 1 with an intestinal perforation).

**Table 3:** Treatment emergent adverse events in radium-223 treated patients according to symptom status

Adverse events^a^	Asymptomatic *N* = 135		Symptomatic *N* = 548	
	Grade 1–2	Grade 3–4	Grade 1–2	Grade 3–4
Any	41 (30)	39 (29)	185 (34)	218 (40)
Anemia^b^	12 (9)	6 (4)	64 (12)	72 (13)
Neutropenia^c^	1 (< 1)	5 (4)	7 (1)	9 (2)
Thrombocytopenia^d^	5 (4)	2 (1)	25 (5)	22 (4)
Constipation	2 (1)	0	23 (4)	6 (1)
Diarrhea	15 (11)	0	59 (11)	4 (< 1)
Nausea	15 (11)	0	74 (14)	1 (< 1)
Vomiting	6 (4)	0	27 (5)	7 (1)
Asthenia	1 (< 1)	0	22 (4)	2 (< 1)
Fatigue	14 (10)	1 (< 1)	39 (7)	12 (2)
General physical health deterioration	0	2 (1)	7 (1)	12 (2)
Weight decreased	9 (7)	1 (< 1)	34 (6)	5 (< 1)
Decreased appetite	10 (7)	0	34 (6)	2 (< 1)
Arthralgia	1 (< 1)	1 (< 1)	20 (4)	1 (< 1)
Back pain	5 (4)	1 (< 1)	24 (4)	19 (3)
Bone pain	8 (6)	3 (2)	75 (14)	26 (5)
Spinal cord compression	0	0	3 (< 1)	19 (3)

Data are number of patients (%) reported in ≥3% in either group and ordered as MedDRA system organ class and preferred terms. Grade 5 TEAEs were reported in 2 (1%) asymptomatic and 32 (6%) symptomatic patients. ^a^Reported as MedDRA preferred terms during the treatment period. Combined MedDRA preferred terms: ^b^anemia and hemoglobin decreased; ^c^neutropenia and neutrophil count decreased; ^d^thrombocytopenia and platelet count decreased. MedDRA, Medical Dictionary for Regulatory Activities

## Discussion

Some clinicians would consider treating patients with mCRPC and asymptomatic disease with radium-223 in their current clinical practice [16]. It may be that administering radium-223 to patients earlier in their disease course, before the onset of severe symptoms and patient clinical deterioration, would lead to improved outcomes. In this iEAP, the safety profile of radium-223 appeared to be better in patients with asymptomatic mCRPC compared with symptomatic disease, with no unexpected adverse events reported in either group. This included fewer TEAEs that were considered to be related to treatment in the asymptomatic group, despite a higher proportion of patients completing 6 radium-223 cycles compared with symptomatic patients. Clinical outcome was also better in radium-223 treated patients with asymptomatic disease who experienced longer overall survival, longer time to disease progression, and a lower risk of SSEs during the study compared with those treated with radium-223 with symptomatic disease. Furthermore, whilst ALP response was similar between the groups, a higher proportion of asymptomatic patients experienced ALP normalization, and PSA responses, compared with symptomatic patients treated with radium-223. Changes in PSA levels are not considered to be a reliable maker for monitoring radium-233 efficacy in this setting [17]. The current findings of a higher PSA response in patients with asymptomatic bone metastases require validation and further investigation in prospective studies.

In the ALSYMPCA study, 513 out of 921 (56%) randomly assigned patients had recorded opioid use at baseline (345 assigned to radium-223 and 168 to placebo). In a subgroup analysis, radium-223 compared with placebo improved overall survival and reduced the risk of initial SSEs during the study, regardless of baseline opioid use [18]. Indeed, radium-223 appeared to be more effective in delaying SSEs in the minimally symptomatic (WHO ladder pain score 0–1) patients who did not require opioid use at baseline (HR: 0.56, 95% CI: 0.39–0.82) compared with those patients with more advanced symptomatic disease (WHO ladder pain score 2–3) who required baseline opioid therapy (HR: 0.72, 95% CI: 0.53–0.98). Furthermore compared with placebo, radium-223 prolonged time to first opioid use and EBRT for pain in the non-opioid group of patients. Radium-223 was well tolerated in patients irrespective of their opioid use at baseline. The authors concluded that pain symptom severity should not be the basis for determining appropriate timing of radium-223 treatment [18].

In this iEAP, patients with asymptomatic disease had more favorable baseline factors that are associated with good prognosis [15, 19, 20], including lower median ALP and PSA levels and lower ECOG PS, suggesting that they had generally less advanced disease than those who had symptoms. Further, the longer time between cancer diagnosis and appearance of bone metastases recorded in the asymptomatic patients may be indicative of a slower disease course in this subgroup of patients. In a separate post hoc analysis of the iEAP, the likelihood of completing radium-223 treatment (receiving 5–6 radium-223 injections) was increased in patients with more favorable prognostic factors at baseline (less pain, low ECOG PS, low PSA level and high hemoglobin level) [21]. In the analysis, overall survival was reported to be longer in patients who received 5–6 injections of radium-223 compared with those who discontinued radium-223 early (received only 1–4 injections of radium-223) [21]. Similar findings were reported from a post hoc analyses of a US EAP and the ALSYMPCA study [22].

Patients treated in this iEAP were generally similar to those currently treated in routine clinical practice, and included chemotherapy-naïve patients, and those who had previously received or were receiving concomitant treatment with abiraterone or enzalutamide [15]. This contrasts with the ALSYMPCA study where patients previously treated with chemotherapy or those who were ineligible for chemotherapy treatment were recruited, but at the time of the ALSYMPCA study abiraterone and enzalutamide were investigational agents and were therefore unavailable [6].

During the treatment period, comparing asymptomatic with symptomatic patients, 21% vs 16% were treated with concomitant abiraterone and 6% vs 5% with concomitant enzalutamide respectively. It is important to note that concomitant treatment of these patients with radium-223 and new hormonal agents may have affected the outcome observed [15]. As similar proportions of asymptomatic vs symptomatic patients received concomitant abiraterone or enzalutamide, we believe that concomitant treatment alone is unlikely to account for the difference in overall survival (medians 20.5 vs 13.5 months) observed between these patient groups.

A phase 3 randomized, double-blind study of radium-223 or placebo, each in combination with abiraterone plus prednisone in chemotherapy-naive patients with asymptomatic or mildly symptomatic mCRPC with bone metastases (ERA 223; NCT02043678) was recently prematurely unblinded. The independent data monitoring committee recommended unblinding the trial due to the observation of more fractures and deaths in the combination treatment arm. Given these results from the ERA 223 trial, the current recommendation is not to combine radium-223 with concomitant abiraterone acetate and prednisone in this asymptomatic patient population [23]. The phase 3 PEACE III trial evaluating radium-223 in combination with enzalutamide versus enzalutamide alone, in patients with mildly symptomatic or asymptomatic mCRPC, is ongoing.

In this single-arm iEAP, the association between symptoms and overall survival confirms the prognostic value of patient symptoms at baseline. Using radium-223 earlier in the disease course, when patients are asymptomatic or minimally symptomatic, may enable patients to complete treatment and optimize treatment outcome compared to symptomatic patients, and therefore may allow sequencing with other life-prolonging therapies.

## Conclusion

In conclusion, use of radium-223 in this group of 135 asymptomatic patients seems to be safe in the setting of this iEAP, however, caution is still warranted in daily clinical practice, as the subgroup size from this study was small, the drug is not approved in this setting, and the final results of prospective studies have to be awaited.

## Additional files

Additional file 1: **Table S1.** Patient disposition according to symptom status. (DOCX 30 kb)

Additional file 2: **Table S2.** SSEs occurring during the study according to symptom status. (DOCX 28 kb)

Additional file 3: **Table S3.** Total-ALP response. (DOCX 29 kb)

Additional file 4: **Table S4.** PSA response. (DOCX 29 kb)

Additional file 5: **Table S5.** Summary of treatment-related adverse events by symptom status. (DOCX 31 kb)

## Abbreviations

ALP: Alkaline phosphatase
BPI-SF: Brief Pain Inventory Short Form
BSoC: Best standard of care
CRPC: Castration-resistant prostate cancer
EBRT: External beam radiation therapy
ECOG PS: Eastern Cooperative Oncology Group performance status
HR: Hazard ratio
iEAP: International early access program
mCRPC: Metastatic castration-resistant prostate cancer
MedDRA: Medical Dictionary for Regulatory Activities
PSA: Prostate specific antigen
SSE: Symptomatic skeletal event
TEAEs: Treatment-emergent adverse events
ULN: Upper limit of normal
