# Supplementary material for: Radium-223 in asymptomatic patients with castration-resistant prostate cancer and bone metastases treated in an international early access program
Source: BMC Cancer. 2019 Jan 7;19:12. doi: 10.1186/s12885-018-5203-y (PMC6322274; doi:10.1186/s12885-018-5203-y)
Supplement: Supplementary file 1 — Table S1. Patient disposition according to symptom status. (DOCX 30 kb) [file 12885_2018_5203_MOESM1_ESM.docx]

**Table A1** Patient disposition according to symptom status

|  | **Asymptomatic *N*=135** | **Symptomatic *N*=548** |
| --- | --- | --- |
| Received all 6 radium-223 injections | 96 (71) | 300 (55) |
| Premature Discontinuation of Treatment | 39 (29) | 248^a^ (45) |
| Death | 0 | 7 (1) |
| Patient withdrawal | 6 (4) | 18 (3) |
| Lost to follow-up | 0 | 1 (<1) |
| Progressive disease | 7 (5) | 53 (10) |
| Physician decision | 2 (1) | 2 (<1) |
| Adverse event not associated with clinical disease progression | 11 (8) | 53 (10) |
| Adverse event associated with clinical disease progression | 10 (7) | 98 (18) |
| Progressive disease - radiological progression | 0 | 3 (<1) |
| Progressive disease - clinical progression | 0 | 2 (<1) |
| Other | 3 (2) | 9 (2) |
| Entered Active Follow-up | 103 (76) | 361 (66) |

Data are number of patients (%).

^a^Discontinuation reasons for three patients not receiving 6 injections were not collected. One patient received 6 injections but had discontinuation reason captured.
