# Supplementary material for: Radium-223 in asymptomatic patients with castration-resistant prostate cancer and bone metastases treated in an international early access program
Source: BMC Cancer. 2019 Jan 7;19:12. doi: 10.1186/s12885-018-5203-y (PMC6322274; doi:10.1186/s12885-018-5203-y)
Supplement: Supplementary file 2 — Table S2. SSEs occurring during the study according to symptom status. (DOCX 28 kb) [file 12885_2018_5203_MOESM2_ESM.docx]

**Table A2** SSEs occurring during the study^a^ according to symptom status

| **SSE category** | **Asymptomatic  *N*=135** | **Symptomatic  *N*=548** |
| --- | --- | --- |
| Any^a^ | 13 (10) | 130 (24) |
| Pathological bone fracture | 6 (4) | 32 (6) |
| Spinal cord compression | 0 | 37 (7) |
| EBRT (for bone pain) | 11 (8) | 94 (17) |
| Tumor-related orthopedic surgical intervention | 2 (1) | 18 (3) |

^a^SSE occurring during the study after the first injection of radium-223.

Data are number of patients (%). EBRT, external beam radiotherapy; SSE(s), symptomatic skeletal event(s).
