# Supplementary material for: Radium-223 in asymptomatic patients with castration-resistant prostate cancer and bone metastases treated in an international early access program
Source: BMC Cancer. 2019 Jan 7;19:12. doi: 10.1186/s12885-018-5203-y (PMC6322274; doi:10.1186/s12885-018-5203-y)
Supplement: Supplementary file 3 — Table S3. Total-ALP response. (DOCX 29 kb) [file 12885_2018_5203_MOESM3_ESM.docx]

**Table A3** Total-ALP response

|  | **Asymptomatic** | **Symptomatic** |
| --- | --- | --- |
| **Safety population, *n*** | **135** | **548** |
| ALP response^a^, *n* (%) | 62 (46) | 259 (47) |
| ALP response by cycle number, *n* (%)^b^ |  |  |
| Cycle 2 | 21 (34) | 120 (46) |
| Cycle 3 | 22 (35) | 80 (31) |
| Cycle 4 | 5 (8) | 36 (14) |
| Cycle 5 | 11 (18) | 10 (4) |
| Cycle 6 | 3 (5) | 10 (4) |
| End of treatment | 0 | 3 (1) |
| **Patients with baseline ALP >ULN, *n*** | **71** | **342** |
| ALP response^a^, *n* (%) | 43 (61) | 212 (62) |
| ALP response by cycle number, *n* (%)^b^ |  |  |
| Cycle 2 | 17 (40) | 106 (50) |
| Cycle 3 | 17 (40) | 65 (31) |
| Cycle 4 | 4 (9) | 25 (12) |
| Cycle 5 | 4 (9) | 7 (3) |
| Cycle 6 | 1 (2) | 6 (3) |
| End of treatment | 0 | 3 (1) |

^a^≥30% confirmed reduction in total-ALP.

^b^Day 1 of the cycle when the first response was observed. Percentages are based on total number of patients with a confirmed response.

ALP, alkaline phosphatase; ULN, upper limit of normal.
