# Supplementary material for: Radium-223 in asymptomatic patients with castration-resistant prostate cancer and bone metastases treated in an international early access program
Source: BMC Cancer. 2019 Jan 7;19:12. doi: 10.1186/s12885-018-5203-y (PMC6322274; doi:10.1186/s12885-018-5203-y)
Supplement: Supplementary file 4 — Table S4. PSA response. (DOCX 29 kb) [file 12885_2018_5203_MOESM4_ESM.docx]

**Table A4** PSA response

|  | **Asymptomatic** | **Symptomatic** |
| --- | --- | --- |
| **Safety population, *n*** | **135** | **548** |
| PSA response^a^, *n* (%) | 29 (21) | 72 (13) |
| PSA response by cycle number, n (%)^b^ |  |  |
| Cycle 2 | 11 (38) | 27 (38) |
| Cycle 3 | 6 (21) | 16 (22) |
| Cycle 4 | 2 (7) | 13 (18) |
| Cycle 5 | 6 (21) | 9 (13) |
| Cycle 6 | 3 (10) | 4 (6) |
| Post cycle 6 visit | 1 (3) | 1 (1) |
| End of treatment | 0 | 2 (3) |
| **Patients with baseline PSA >ULN, *n*** | **129** | **514** |
| PSA response^a^, *n* (%) | 28 (22) | 65 (13) |
| PSA response by cycle number, *n* (%)^b^ |  |  |
| Cycle 2 | 10 (36) | 25 (38) |
| Cycle 3 | 6 (21) | 15 (23) |
| Cycle 4 | 2 (7) | 10 (15) |
| Cycle 5 | 6 (21) | 8 (12) |
| Cycle 6 | 3 (11) | 4 (6) |
| Post cycle 6 visit | 1 (4) | 1 (2) |
| End of treatment | 0 | 2 (3) |

^a^≥30% confirmed reduction in PSA.

^b^Day 1 of the cycle when first response was observed. Percentages are based on total number of patients with a confirmed response.

PSA, prostate-specific antigen; ULN, upper limit of normal.
