# Supplementary material for: Radium-223 in asymptomatic patients with castration-resistant prostate cancer and bone metastases treated in an international early access program
Source: BMC Cancer. 2019 Jan 7;19:12. doi: 10.1186/s12885-018-5203-y (PMC6322274; doi:10.1186/s12885-018-5203-y)
Supplement: Supplementary file 5 — Table S5. Summary of treatment-related adverse events by symptom status. (DOCX 31 kb) [file 12885_2018_5203_MOESM5_ESM.docx]

**Table A5** Summary of treatment-related adverse events by symptom status

| **Adverse events**^a^ | **Asymptomatic *N*=135** | | | **Symptomatic *N*= 548** | | |
| --- | --- | --- | --- | --- | --- | --- |
|  | **Grade 1–2** | **Grade 3–4** | **Grade 5** | **Grade 1–2** | **Grade 3–4** | **Grade 5** |
| Any | 29 (21) | 9 (7) | 0 | 169 (31) | 72 (13) | 2 (<1) |
| Anemia^b^ | 8 (6) | 3 (2) | 0 | 33 (6) | 28 (5) | 0 |
| Neutropenia^c^ | 1 (<1) | 3 (2) | 0 | 5 (<1) | 6 (1) | 1 (<1) |
| Thrombocytopenia^d^ | 5 (4) | 2 (1) | 0 | 20 (4) | 13 (2) | 0 |
| Diarrhea | 12 (9) | 0 | 0 | 46 (8) | 3 (<1) | 0 |
| Intestinal perforation | 0 | 0 | 0 | 0 | 0 | 1 (<1) |
| Nausea | 9 (7) | 0 | 0 | 52 (9) | 0 | 0 |
| Vomiting | 4 (3) | 0 | 0 | 18 (3) | 4 (<1) | 0 |
| Fatigue | 9 (7) | 0 | 0 | 22 (4) | 4 (<1) | 0 |
| Weight decreased | 3 (2) | 0 | 0 | 15 (3) | 0 | 0 |
| Decreased appetite | 5 (4) | 0 | 0 | 18 (3) | 0 | 0 |
| Bone pain | 3 (2) | 1 (<1) | 0 | 30 (5) | 4 (<1) | 0 |

Data are number of patients (%) reported in ≥3% and all grade 5 in either group. ^a^Reported as MedDRA preferred terms during the treatment period. Combined MedDRA preferred terms: ^b^anemia and hemoglobin decreased; ^c^neutropenia and neutrophil count decreased; ^d^thrombocytopenia and platelet count decreased. MedDRA, Medical Dictionary for Regulatory Activities; TEAE, treatment emergent adverse event.
